# Prevalence of Depression and Associated Factors among Patients with Type 2 Diabetes Attending the Diabetic Clinic at a Tertiary Care Hospital in Sri Lanka: A Descriptive Study

**DOI:** 10.1155/2019/7468363

**Published:** 2019-02-03

**Authors:** Maulee Hiromi Arambewela, Noel P. Somasundaram, Hettiarachchige Buddhi Pradeep Ranjan Jayasekara, Mahesh P. Kumbukage

**Affiliations:** ^1^Department of Diabetes and Endocrinology, National Hospital of Sri Lanka, Colombo, Sri Lanka; ^2^Department of Physiology, Faculty of Medical Sciences, University of Sri Jayawardenepura, Nugegoda, Sri Lanka; ^3^Ministry of Health, Colombo, Sri Lanka

## Abstract

**Background:**

Research focusing on the psychological aspect of diabetes is limited in Sri Lanka.

**Aim:**

Determine the prevalence of depression among patients with type 2 diabetes mellitus (T2DM) attending an out-patient clinic at a tertiary care hospital in Colombo, the capital of Sri Lanka.

**Methods:**

A descriptive cross-sectional study carried out among patients diagnosed with T2DM. Pregnant and patients with a prior psychiatric history were excluded. Depression assessed using validated Sinhala and Tamil version of the Beck's Depression Index. Sociodemographic data and health related data were obtained from interviewer-based questionnaires and health records.

**Results:**

Of the 3000 patients, 72.7% were female. Mean age was 58.3 ±10.3 years and mean duration of diabetes 10.8 ± 7.3 years. Percentage of depression was 5.9% in the entire patient population with mild, moderate, and severe depression in 4.0%, 1.6%, and 0.3%, respectively. In multiple logistic regression, depression was significantly associated with female gender (OR 2.63, 95% CI 1.26-5.46; P=0.009), living without a spouse (single/divorced/widowed) (OR 1.83, 95% CI 1.12-2.98; P=0.01), lower education level (OR 1.92, 95% CI 1.14-3.22; P=0.01), and peripheral neuropathy (OR 1.79, 95% CI 1.00-3.18; P=0.04). Only 13.3% of the respondents said that doctors have ever inquired to their mental well-being.

**Conclusion:**

Depression was low in prevalence comparative to neighboring countries in the region. Patients were much more affected by the social factors than disease related factors. An individualized holistic approach taking psychosocial issues to consideration should be focused in the comprehensive plan of management.

## 1. Background

The world is facing an unprecedented epidemic of diabetes with the current global prevalence estimated to be 285 million and projection rates expected to rise over 438 million by the year 2030 [1]. The major bulk of this is borne in Asia. In Sri Lanka, statistics reveal alarming figures of one in five adults being diabetic or prediabetic and one-third of those with diabetes being undiagnosed [2].

Any chronic illness will have a negative impact on its sufferer. Diabetes with comorbid depression can have poor self-care, treatment adherence and glycaemic control as well as increased morbidity and mortality [3]. Diabetes and depression can have a bidirectional relationship. Patients with diabetes are liable to become depressed due to the chronic nature of the disease and its numerous complications while diabetes can arise in depression due to an increase in counter regulatory hormones. Study done in relation to diabetes, depression, and functional disability among adults in the United States concluded that individuals with diabetes and comorbid depression have higher odds of functional disability when compared with individuals with either diabetes or major depression alone [4].

Data on depression among diabetics in Sri Lanka is scanty. A cross-sectional population-based survey by Ball et al. done in 2010 demonstrated a 6.1% prevalence of life time-ever depression among the population in Colombo district [5]. This study used a Composite International Diagnostic Interview where depressive disorder was diagnosed by Diagnostic and Statistical Manual of Mental Disorders, 4th Edition, Text Revision (DSM IV-TR) criteria. In another study, the prevalence of depression among newly diagnosed patients with diabetes in Sri Lanka revealed that 13.4% had mild depression and 15.6% had moderate depression at the time of diagnosis and highlighted the importance of screening patients with diabetes for depressive disorders [6]. However, this study was done on a small population of 186 patients. The diagnosis of depressive disorder were done categorically following a clinical interview by the consultant psychiatrist, based on International Classification of Disease, Tenth Edition (ICD 10 ) criteria.

Up to now there had been no other published data related to depression and diabetes in Sri Lanka. Depression in the diabetes population has been associated with potential sociodemographic and clinical factors. Ageing, ethnicity, socioeconomic status, education level, and unemployment were important correlates for depression among people with diabetes [7].

Most studies done in Sri Lanka focus mainly on the physical aspect of this disease. However, being a chronic and debilitating disease, it has considerable impact on the sufferer's psychosocial well-being and it is time that the focus shifted to a more holistic approach to optimize care. Therefore, we set out to study the prevalence of depression and its associations among patients with type 2 diabetes attending a tertiary care hospital in Sri Lanka.

## 2. Methods

### 2.1. Aim

The aim of this study was to assess the prevalence of depression as defined by a score of 17 or more in the Beck's Depression Index (BDI) and associated factors among a large cohort of patients with type 2 diabetes attending a tertiary care hospital in Sri Lanka.

### 2.2. Study Setting and Population

This was a descriptive cross-sectional single centre study carried out at the National Hospital Sri Lanka during the period of 01/01/2016 to 31/7/2016. National Hospital is the biggest hospital in Sri Lanka with a bed strength of more than 3000. Around 16000 patients are registered in the diabetic clinic and around 400 patients a day attend the clinic to seek care. It caters mainly to diabetic patients in Colombo and its suburbs.

### 2.3. Inclusion and Exclusion Criteria

Patients who were aged over 18 years attending the diabetic clinic diagnosed with diabetes according to the American Diabetic Association (ADA) criteria for at least 3 months were systematically sampled using clinic attendance register. Pregnant patients and patients with a prior psychiatric history were excluded. Patients with psychiatric disorder were excluded as this study attempted to evaluate depression as a secondary condition to diabetes. Criteria for prior psychiatric history included taking psychiatric medication at present or being diagnosed with a mental disorder.

### 2.4. Study Instruments

Depression was assessed by Beck's Depression Inventory (BDI) which is a 21-question multiple-choice self-report inventory, one of the most widely used instruments for measuring the severity of depression. Previous studies have demonstrated BDI as an accurate tool in screening for depression among patients with type 2 diabetes [8]. Being a diverse and multicultural country, Sri Lanka is home to many religions, ethnic groups, and languages. The three main languages used by Sri Lankans are Sinhala, Tamil, and English. Therefore, data was collected by specially trained data collectors in these three languages. The Sinhala version has been previously validated in Sri Lanka [9]. The English version of the Beck's Depression Index was translated in to Tamil by academic linguists. The Tamil translation was then back translated by a second group of linguists. The process of translation and back translation was repeated until the back translation matched the English version which was the control. A pilot study was carried out among fifty Tamil speaking patients. The questionnaire was administered to all fifty patients and the same group of patients were separately clinically evaluated by a psychiatrist to determine the presence or absence of depression. A sensitivity of 74% and a specificity of 85% were obtained at the cut off of a score of 17. A score of over 17 out of 63 in the BDI was considered in keeping with clinically significant depression. Scores between 17 and 20 was classified as mild depression, while scores between 21-30 and >30 were classified as moderate and severe depression respectively. A subset of the population was interviewed by a consultant psychiatrist to cross validate the questionnaire. This revealed no marked difference between the results from the questionnaire and the diagnosis of the psychiatrist.

A structured questionnaire was administered by trained data collectors conversant in all three languages to obtain information on sociodemographic, financial, educational, and occupational data.

A data sheet on macro- and microvascular complications and current medication was filled in by medical officers working in the diabetic clinic by going through the patient's medical records.

### 2.5. Ethical Issues

Ethical clearance was obtained from the ethical review committee of the University of Colombo prior to initiation of the study. Administrative approval was obtained from the National Hospital representatives to carry out the study. Participation was entirely voluntary and written informed consent was obtained from the participants.

### 2.6. Statistical Analysis

Statistical analysis was performed using the Statistical Package for Social Sciences (SPSS) version 20. Frequencies of all variables were analysed using descriptive statistics. Data was reported as mean ± SD and percentages. Associations for predictor variables with depression were examined initially with bivariate analysis and then with multiple logistic regression to adjust for potential confounding factors using backward stepwise method. Significant level was set at 5%. Results were expressed as odds ratios (OR) and 95% confidence intervals (CI). P value <0.05 was considered statistically significant.

The final multivariable model consisted of age, duration of diabetes, gender, civil status, income, occupation, level of education, family type, peripheral neuropathy, retinopathy, stage of CKD, diabetic foot, PVD, IHD, stroke, BMI, glycemic control, insulin use, pill count, and alcohol use. These variables were included in the final model due to known associations with depression in literature.

## 3. Results

Out of the three thousand patients studied in this patient population, 2180 were females (72.7%). Mean age was 58.3 (SD±10.3) years with 43.3% of the population being over the age of 60 years. Mean duration of diabetes was 10.8 (SD ± 7.3) years. Majority of the patients (68.3%) were from nuclear families and 71.5% of the study population were married. Almost half of the respondents (49.6%) were retired and 75.6% of the study population had a monthly income less than Sri Lankan Rupees 30,000/=. Regarding level of education, 4.3% have never been to school while the majority of 78.2% have had a secondary education. Sociodemographic data of the respondents is summarized in Table 1.

Mean HbA1c level was 8.34%  ± 2.57 with only 28.5% of patients having their HbA1c level <7% which is the ideal level for good glycemic control. Mean Basal Metabolic Index (BMI) of the population was 25.6+/-6.6 with 54% being over the cutoff for obesity. High blood pressure was seen in 77.6% of the patients. Percentage of depression was 5.9% in the entire patient population with mild, moderate, and severe depression seen in 4.0%, 1.6% and 0.3%, respectively. Only 13.3% of the respondents said that doctors have ever inquired to their mental well-being and problems encountered when managing their diabetes. Baseline clinical data inclusive of prevalence of macro- and microvascular complications are summarized in Table 2.

Bivariate analysis for associated variables with depression using chi square showed significant associations with female sex (Unadjusted Odds Ratio (UOR)= 4.56, 95% CI=2.22-6.87; P<0.0001), not being married (single/divorced/widowed). UOR=2.44*¸*95% CI =1.79-3.33; P<0.0001), low income (UOR=2.21, 95% CI=1.41-3.46; P<0.001), low education level (UOR=2.45, 95% CI=1.77-3.40; P<0.001), occupational status (UOR=1.57, 95% CI=1.08-2.28; P=0.01), being in an extended family (UOR= 1.43, 95% CI=1.13-2.86; P=0.034), peripheral neuropathy (UOR=1.4, 95% CI=1.03-1.95; P=0.048), current alcohol use (UOR= 1.34, 95% CI= 1.04-1.95; P=0.045), and retinopathy (UOR=1.50, 95% CI=1.13-2.08; P=0.032) (Table 3).

However multiple logistic regression analysis showed significant associations with female gender (AOR = 2.63, 95% CI 1.26-5.46), being not married (single/widowed/divorced) (AOR = 1.83, 95% CI 1.12–2.98), education level below primary level (AOR = 1.92, 95% CI 1.14-3.22), and peripheral neuropathy (AOR = 1.79, 95% CI 1.01-3.18) (Table 4).

There were no associations with sociodemographic variables such as age, family type, income, occupation, and disease variables such as stage of chronic kidney disease, diabetic foot disease, retinopathy, peripheral vascular disease, stroke, ischemic heart disease, duration of diabetes, BMI, glycemic control, use of insulin, and pill count.

## 4. Discussion

This was a large cross-sectional study carried out among 3000 patients with the aim of assessing the prevalence of depression and associated factors among patients with T2DM attending the largest tertiary care hospital situated in the country's capital city of Colombo. The prevalence of depression in this population was 5.9 % with 4.0%, 1.6%, and 0.3% having mild, moderate, and severe depression, respectively. This was in stark contrast to data from other countries in the region as well as the world. The DAWN study which was a large cross-sectional study carried out among 5104 patients from 13 different countries including Asia, Australia, Europe, and North America concluded that 41% of the adults with type 2 diabetes had poor psychosocial well-being. Asia was represented by India and Japan in this study [10]. Studies done in Malaysia, in a group of 169 patients with type 2 diabetes, reported a prevalence of anxiety in 31.4% and depression in 40.3% [11].

Studies done on depression among diabetics are scarce in Sri Lanka. A study done by Amarasinghe et al. on 186 patients with newly diagnosed diabetes in the Chilaw District General Hospital reported that a quarter of the study population had depression [6]. Such high degree of depression may be attributed to the initial stage of diagnosis when patients become aware of the potential complications in future, psychosocial demands, and life style changes required. Furthermore, depression was diagnosed categorically following a clinical interview by the consultant psychiatrist, based on ICD 10 criteria and categorized as mild, moderate, and severe. These categories differ slightly from BDI used in this study as the ICD-10 seems more sensitive to the mild range of the depression spectrum compared to the BDI which uses the DSM IV criteria [12]. Therefore differences in study tool and study population can contribute to the discrepancies in the prevalence rates. Other similar studies have been done in patients with chronic medical illnesses in Sri Lanka. Depression was reported at a prevalence of 27.9% in a study conducted among 140 patients with chronic renal failure in the North Central Province of Sri Lanka [13]. This study used a Structured Clinical Interview for DSM disorders (SCID) for diagnosing depression. Prevalence of depression was 38.4% in 211 patients who presented to a tertiary cardiology centre in the central province following acute myocardial infarction [14].

A combination of tools including validated Sinhala version of the BDI, Zung Self-Rating Depression Scale (SDS), and consultant psychiatrist's clinical assessment using the Diagnostic Criteria for Research was used in the previous study while the depression scale used in Centre for Epidemiologic studies was used as the research tool in the latter. Another study which looked into the depressive disorders among patients attending the out-patient department in a tertiary care hospital in Colombo reported an overall prevalence of 22.4%, with females being more affected than males (25.4% versus 18.7%) and significant association between pain related presenting complaint [15]. All these studies show that there is a wide variation in the rate of depression among various populations suffering from chronic illnesses. This needs to be taken in context that the variety of different tools used for the diagnosis of depression may have contributed in part to the variation in prevalence.

The only study looking in to the population prevalence of depression in Sri Lanka by Ball et al. demonstrated a 6.1% prevalence of life time-ever depression among the population in Colombo district [5]. Even though this study was carried out during the height of the civil war population prevalence of depression in Colombo was low in comparison to other Western countries. This study used a Composite International Diagnostic Interview where depressive disorder was diagnosed by DSM IV criteria by lay interviewer-based questionnaire. Presence or absence of depression was based on a dimensional scale. The prevalence of depression in our study population was keeping with the population prevalence. This low rate of depression may be because these were patients suffering from long-standing diabetes (the mean duration of disease 10 years) and that the majority were not yet affected with the more debilitating forms of chronic complications limiting functional ability.

An important factor contributing to the low prevalence of depression in this study population when compared to other Sri Lankan studies may be attributed to exclusion of patients with prior psychiatric disorders. Therefore a proportion of patients with diabetes who developed depression as a result of the disease per se would have been excluded.

Females were significantly found to be more depressed than males in this study population. Many such studies done on similar populations have revealed similar results [16, 17]. It may be in keeping with epidemiologic prevalence data that reveal women are approximately 1.7 times as likely as men to report a lifetime history of major depressive episodes [18]. It could also be attributed to the sociocultural roles played by the women in these countries including responsibilities at work and home, child care, psychological aspects, and lack of social support.

Depression was found to be significantly associated with lower education level. Higher education is a protective factor against depression in diabetics. Education provides better understanding of the disease and its complications and leads to better compliance and self-care [19].

Evidence suggests that civil status plays a role with those being married having less rates of depression [18]. This was evident in our population as well. Depression was significantly lower among married patients in contrast to those who were single/divorced or widowed. As diabetes is a disease which requires social support for the individual, it is the common school of thought that patients in nuclear families may tend to be more depressed than the patients in extended families. This was in contrast in this study population where depression was higher among patients in extended families. This may be due to the numerous challenges faced by the extended family living in a highly urbanized setting. Unemployment has been liked positively with depression among diabetes in previous studies [20]. However in the current study, occupation, income, and family type were found to have significant associations with depression in bivariate analysis but were not so following multiple logistic regression. This is most likely due to confounding factors.

Advancing age is usually associated with depression and more so in patients with diabetes [21]. In the current study, no significant association between age and depression was found.

Many studies have reported on the strong relationship between obesity and depression [22]. The alarming rise in obesity goes hand in hand with the increase in prevalence of diabetes. Altered body image associated with obesity, development of diabetes, and its comorbidities and depression all fall into a vicious cycle. In the study sample, 56.6% of the study population had a BMI value of more than 25. However an association was not observed in our study.

Diabetes associated macro- and microvascular disease is known to have adverse psychological effects among patients with diabetes [23]. Surprisingly depression was only found to have a significant association with neuropathy in our patient population. Another important disease variable is poor glycemic control which can result in depression Reports regarding the association between glycaemic control and depression are conflicting [24, 25]. Dysglycemia may result in depression and vice versa. However, there was no difference in HbA1c levels among our patients with and without depression. The fact that these patients were having chronic long-standing diabetes and that the majority were not affected with the more severe form of the chronic complications which limit functional disability may be a reason for the low rate of depression.

## 5. Conclusion

Depression was found to be surprisingly low at a prevalence of 5.9% in this patient population, being almost similar to the population prevalence. This was in stark contrast to other neighboring countries in the region and the rest of the world where prevalence ranged from 20-40%. The risk factors for depression were female gender, low level of education, not being married, and neuropathy suggesting patients were far more affected by the social context they live in and that concern for diabetes and its care takes backstage. Furthermore, doctors' focus on psychosocial health of the patients was reported as low.

As diabetes poses many challenges to individuals at different levels, including psychosocial factors, health care providers need to be trained to understand the impact of these on the disease. An individualized, holistic approach to medical care inclusive of mental and social well-being should be the focus on a comprehensive care plan for each patient living with diabetes.

*Strengths and Limitations of the Study*. This was a large study carried out on 3000 patients. Patients were included irrespective of their literacy level as data was collected from interviews and from health records without relying on self-responses on diabetes related data.

An important limitation is exclusion of previously diagnosed psychiatric disorder patients, thus limiting the inclusion of patients with diabetes who had already been diagnosed with depression. However the authors believe that inclusion of such patients would have hindered the ability to arrive at a reliable conclusion since most psychiatric disorders will have comorbid or secondary depression.

Another important limitation was the diagnosis of depression in the study population. The gold standard method of diagnosis is by a psychiatrist following a psychiatric diagnostic interview. In our study we used the BDI which is a widely accepted and validated screening tool which should be ideally self-administered. However, due to various practical issues the questionnaires were filled in by specially trained data collectors. To minimize this limitation, we cross validated the BDI in a subset of the patient population with the gold standard method of diagnosis. This however did not yield a significant difference. The lack of a control group of patients from the general population without diabetes and failing to acknowledge other concurrent medical illnesses were limitations.

## Figures and Tables

**Table 1:** Sociodemographic characteristics of the respondents.

**Characteristic**	**Number**	**Percentage**
**Gender**		
Male	820	27.3
Female	2180	72.7
**Age**		
Less than 60	1702	56.7
More than 60	1298	43.3
**Family type**		
Nuclear	2048	68.3
Extended	952	31.7
**Civil Status**		
Married	2145	71.5
Single	134	4.5
Divorced	60	2.0
Widowed	657	21.9
Other	4	0.1
**Level of education**		
No schooling	129	4.3
Primary Education (Grade 1-5)	446	14.9
Secondary Education	2347	78.2
Tertiary Education	78	2.6
**Level of income**		
< Rs 30,000	2268	75.6
>Rs 30,000	732	24.4
**Occupation**		
Managers and Professional	110	3.7
Non-Professionals	764	25.5
Housewives	309	10.3
Retired	1489	49.6
Unemployed	328	10.9

**Table 2:** Clinical health related indices of the patient population.

**Disease characteristic **	**Number**	**Percentage**
**Duration of diabetes **		
>10 years	1307	43.6
**BMI **		
>25	1722	56.6
**Glycemic control HbA1c (n=1796) **		
HbA1c >7%	1284	71.5
**Hypertension **	2328	77.6
**Macrovascular disease**		
Ischemic heart disease	308	10.5
Stroke/TIA	33	1.1
Peripheral vascular disease	140	4.7
**Diabetic Retinopathy**	863	28.8
**Diabetic Nephropathy(n=884)**	436	49.3
**CKD Staging**		9.8
Normal	242	
Stage 1	436	17.6
Stage 2	1052	42.5
Stage 3	666	26.9
Stage 4	53	2.1
Stage 5	25	1.0
**Diabetic Neuropathy**	1879	62.6
**Diabetic foot **	78	2.6
**Insulin Use**	832	27.7
**Polypill (Pills>4)**	1260	42.0

**Table 3:** Bivariate analysis using chi square showing the risk factors associated with depression.

**Risk factor **	**Adjusted Odds Ratio (95**%**CI)**
**Gender **	
Females	4.56 (2.22-6.87)
males	1
**Civil status **	
Single/ widowed/divorced	2.44 (1.79-3.33)
Married	1
**Education**	
Primary and below	2.45 (1.77-3.40)
Secondary and upper	1
**Peripheral neuropathy**	
Yes	1.42 (1.03-1.95)
No	1
**Occupational Status**	
Unemployed	1.57 (1.08- 2.28)
Employed	1
**Income Level**	
< Rs 30,000	2.21 (1.41- 3.46)
>Rs 30,000	1
**Current Alcohol Use**	
Yes	1.34 (1.04-1.95)
No	1
**Retinopathy**	
Yes	1.50 (1.13 – 2.08)
No	1
**Family Status**	
Extended Family	1.43(1.13- 2.86)
Nuclear Family	1

**Table 4:** Multiple logistic regression analysis showing the risk factors associated with depression.

**Risk factor **	**Adjusted Odds Ratio (95**%**CI)**	**P value**
**Gender **		
Females	2.63 (1.26-5.46)	0.009
Males	1	

**Civil status **		
Single/ widowed/divorced	1.83 (1.12-2.98)	0.01
Married	1	

**Education**		
Primary and below	1.92 (1.14-3.22)	0.01
Secondary and upper	1	

**Peripheral neuropathy**		
Yes	1.79 (1.01-3.18)	0.047
No	1	

## Data Availability

The data used to support the findings of this study are included within the article.

## Abbreviations

T2DM: Type 2 diabetes mellitus
OR: Odds ratio
CI: Confidence interval
ADA: American Diabetic Association
BDI: Beck's depression inventory
SPSS: Package for Social Sciences
BMI: Basal metabolic index
CKD: Chronic kidney disease
TIA: Transient ischemic attack.
